# Viral–Gut Microbiota Interactions: Implications for the Gut Mucosa in Inflammatory Bowel Disease

**DOI:** 10.3390/ijms27198711

**Published:** 2026-09-29

**Authors:** Yonghong Wu, Lei Hou, Yue Li, Jiao Li, Xue Hua, Yan He, Sujuan Ding, Jun Fang

**Affiliations:** 1 College of Bioscience and Biotechnology, Hunan Agricultural University, Changsha 410128, China; wuyonghong@stu.hunau.edu.cn (Y.W.); liyue@stu.hunau.edu.cn (Y.L.); lijiao@stu.hunau.edu.cn (J.L.); huaxue@stu.hunau.edu.cn (X.H.); hya@stu.hunau.edu.cn (Y.H.); 2 Yuelushan Laboratory, Changsha 410128, China; houlei@csmadik.com.cn

**Keywords:** inflammatory bowel disease, gut virome, bacteriophages, phageome, gut microbiota, intestinal barrier, virus–bacteria interactions

## Abstract

Inflammatory bowel disease (IBD), including ulcerative colitis (UC) and Crohn’s disease (CD), arises from complex interactions among genetic susceptibility, immune dysregulation, environmental factors, and the gut microbiome. Bacterial dysbiosis has been extensively studied, whereas the gut virome—comprising eukaryotic viruses and bacteriophages—remains less understood. Viruses can affect the intestinal barrier directly by infecting epithelial and immune cells, or indirectly by reshaping bacterial communities and host immunity. However, current evidence is dominated by cross-sectional associations and animal models, and whether virome alterations drive or result from intestinal inflammation remains unresolved. In this review, we provide a focused overview of intestinal barrier components relevant to IBD, synthesize human and animal evidence on IBD-associated viruses, and distinguish direct viral effects, microbiota-mediated effects, and bidirectional virus–microbiota interactions. We highlight bacteriophage–bacteria interactions as a central and potentially causal axis, discuss UC- and CD-specific differences, and outline causality gaps, search methodology, and emerging therapeutic strategies. Finally, we propose key unresolved questions and future research directions.

## 1. Introduction

Inflammatory bowel disease (IBD), comprising ulcerative colitis (UC) and Crohn’s disease (CD), is a chronic immune-mediated disorder with increasing global prevalence [1,2,3]. Although immune dysregulation, genetic susceptibility, and environmental factors are central to IBD pathogenesis, the gut microbiome has emerged as a key modifier of disease onset and progression [2,3]. Bacterial dysbiosis in IBD is characterized by reduced diversity, loss of beneficial obligate anaerobes, and expansion of facultative pathobionts [4,5,6,7,8]. In contrast, the gut virome—including eukaryotic viruses and bacteriophages—has received comparatively less attention, largely because of technical challenges in viral sequencing, classification, and culture [9,10,11,12].

Recent advances in metagenomics and virome analysis have revealed that intestinal viruses regulate microbial ecology, host immunity, and intestinal homeostasis, and may contribute to IBD [9,10,11,12]. However, the current literature often conflates three distinct relationships: (i) direct viral effects on host epithelial or immune cells; (ii) microbiota-mediated effects of viral infection; and (iii) bidirectional virus–microbiota interactions. This distinction is essential because detecting a virus in inflamed mucosa does not prove that the microbiota mediates the observed barrier injury.

Therefore, the central question of this review is: to what extent do gut viruses, alone or through interactions with bacterial microbiota and bacteriophages, contribute to intestinal barrier dysfunction in IBD, and what is the evidence for causality? We first summarize barrier components relevant to IBD, then synthesize human and animal evidence on IBD-associated viruses. We distinguish direct viral effects, microbiota-mediated effects, and bidirectional virus–microbiota interactions, and we highlight bacteriophage–bacteria interactions as one of the most direct and biologically meaningful forms of virus–microbiota interaction. Animal and livestock viruses are discussed only as mechanistic barrier-disruption models and are explicitly separated from viruses clinically implicated in human IBD. Finally, we identify unresolved questions regarding causality, biomarkers, UC/CD differences, and therapeutic translation.

### Scope, Conceptual Framework, and Search Strategy

This review addresses a central question: in IBD, how do enteric viruses and bacteriophages influence intestinal mucosal barrier function directly, through the bacterial microbiota, or via bidirectional virus–microbiota interactions? We distinguish three non-mutually exclusive levels of evidence: (i) direct viral effects on epithelial, immune, or acellular barrier components; (ii) microbiota-mediated effects, in which viral infection alters bacterial communities or their metabolites, which then affect the barrier; and (iii) bidirectional virus–microbiota interactions, in which bacteria or phages reciprocally modulate viral infection, persistence, or immunogenicity. We also grade evidence as human clinical association, human interventional/causal evidence, animal model, and in vitro mechanistic evidence.

We searched PubMed, Web of Science, and Scopus for articles published up to 2026 using combinations of terms including inflammatory bowel disease, ulcerative colitis, Crohn’s disease, gut virome, bacteriophage, phageome, gut microbiota, intestinal barrier, tight junction, mucus, antimicrobial peptide, secretory immunoglobulin A, Epstein–Barr virus, cytomegalovirus, norovirus, SARS-CoV-2, and animal models. Studies were prioritized if they directly addressed human IBD or intestinal mucosal barrier mechanisms; animal and in vitro studies were included when they provided mechanistic insights. Reference lists of relevant reviews were also screened. Studies using livestock or laboratory pathogens without established human IBD relevance are discussed separately as mechanistic barrier-disruption models.

## 2. Clinical and Pathophysiological Context of IBD

### 2.1. UC and CD: Distinct but Overlapping Diseases

UC is characterized by continuous colonic inflammation, typically presenting with bloody diarrhea, mucus, urgency, and left lower quadrant pain [13]. CD features segmental, transmural inflammation, often with non-bloody diarrhea, right lower quadrant pain, fistulae, abscesses, and perianal disease [14,15]. Extraintestinal manifestations and colitis-associated colorectal cancer reflect systemic immune activation and long-standing mucosal injury [16,17,18,19,20]. Importantly, UC and CD differ in anatomy, immune phenotype, and barrier involvement; therefore, findings should not be generalized across IBD without specifying the subtype.

### 2.2. Convergent Pathophysiological Mechanisms

IBD pathogenesis involves genetic susceptibility, environmental triggers, immune dysregulation, and gut microbial dysbiosis converging on the intestinal mucosal barrier [7,21,22,23]. Genetic variants in *NOD2*, *ATG16L1*, and *IL23R* impair microbial sensing, autophagy, and epithelial integrity [7,21]. Dietary factors, smoking, antibiotics, and enteric viral infections reshape the gut ecosystem and may trigger flares in predisposed individuals [22,23]. In IBD, bacterial diversity is reduced, beneficial obligate anaerobes such as *Faecalibacterium prausnitzii* are depleted, and facultative pathobionts including adherent-invasive *Escherichia coli* expand [7,8]. Critically, bacterial dysbiosis is interwoven with enteric virome alterations, including bacteriophage compositional shifts and eukaryotic viral infections [24,25,26,27,28,29,30]. These interactions may form a self-amplifying loop in which viral activity reshapes the microbial landscape, which in turn facilitates viral persistence and mucosal invasion. The genetic, environmental, and immunological determinants of IBD therefore converge on a dysregulated microbiota–virome axis, with barrier dysfunction as a central pathogenic event.

## 3. Intestinal Barrier Components and Dysfunction in IBD

### 3.1. Barrier Components Relevant to Virus–Microbiota Interactions

The intestinal barrier is a multilayered system composed of intestinal epithelial cells (IECs), intercellular junctional complexes, mucus, antimicrobial peptides (AMPs), secretory immunoglobulin A (sIgA), and mucosal immune cells [31,32,33,34,35,36,37,38,39]. IECs form a single epithelial layer and continuously sense luminal microorganisms through pattern-recognition receptors (PRRs) [31]. Tight junctions (TJs), composed of claudins, occludin, and zonula occludens (ZO) proteins, regulate paracellular permeability, whereas adherens junctions (AJs), centered on E-cadherin–catenin complexes, maintain mechanical cohesion [32,33,34]. Goblet cells secrete MUC2 mucin, which forms the mucus layer and spatially segregates microorganisms from the epithelium [35,36]. Paneth cells secrete α-defensins, lysozyme, REG3, and secretory phospholipase A2, shaping microbial colonization [38,39]. sIgA limits microbial adhesion and promotes immune exclusion [37]. Together, these components maintain intestinal homeostasis and provide the foundation for balanced host–microbiota interactions.

### 3.2. Barrier Dysfunction in IBD

A hallmark of IBD is junctional disassembly, characterized by disruption of TJ and AJ structural and functional integrity [34,40]. Pro-inflammatory cytokines, particularly TNF-α, IFN-γ, and IL-13, activate myosin light-chain kinase (MLCK)-dependent cytoskeletal contraction, leading to redistribution, internalization, or degradation of ZO-1, occludin, claudins, JAMs, and E-cadherin [41,42,43]. Persistent inflammation also promotes epithelial apoptosis, necroptosis, and pyroptosis while impairing epithelial restitution [44]. Consequently, luminal bacteria, PAMPs, toxins, and dietary antigens traverse the epithelial barrier and access the lamina propria, triggering sustained immune activation. In parallel, goblet cell depletion, defective MUC2 biosynthesis, Paneth cell dysfunction, and impaired sIgA coating weaken the biochemical barrier [45,46,47,48,49,50]. These alterations permit microorganisms to breach the mucus interface and amplify inflammatory responses. Thus, barrier dysfunction and immune dysregulation reinforce one another through a positive feedback loop.

### 3.3. Gut Bacterial Dysbiosis as Context

The host and gut bacteria maintain a delicate equilibrium. In IBD, this equilibrium is disrupted, resulting in reduced bacterial diversity, decreased short-chain fatty acid (SCFA) synthesis, and expansion of pro-inflammatory microorganisms such as adherent-invasive *Escherichia coli* and sulfate-reducing bacteria [51,52,53]. Fungal dysbiosis may also elevate IBD risk by altering bacterial composition or stimulating pro-inflammatory cytokines [54,55,56]. Dysbiosis activates PRRs and promotes local inflammation, impairing gut barrier function [57]. Recent machine learning-based analyses have identified disease-specific microbial signatures, which may facilitate future investigations into virus–bacteria interactions and their contributions to intestinal barrier dysfunction [58].

## 4. Gut Viruses Associated with IBD

### 4.1. Epstein–Barr Virus

Epstein–Barr virus (EBV) is a γ-herpesvirus that establishes lifelong latent infection in B lymphocytes and can reactivate, particularly under immunosuppression [59]. EBV infection has been associated with IBD, with EBV DNA detected in the intestinal mucosa of 79.4% of patients in one clinical study [60]. However, this estimate comes from a single cohort and should not be generalized. EBV has been linked to lymphoplasmacytic infiltration and, in immunosuppressed patients, to EBV-associated colitis and lymphoproliferative disorders [61,62]. Mechanistically, EBV can infect primary human colon epithelial cells and stimulate pro-inflammatory cytokines, ERK1/2 signaling, DNA repair dysregulation, and autophagy impairment [63]. EBV DNA activates endosomal TLR signaling and enhances IL-17A, IFN-γ, and TNF-α in experimental colitis [64,65,66]. Drosophila models also suggest that EBV DNA can exacerbate inflammatory responses [67]. Importantly, most human evidence remains associative, and reverse causation—inflammation or immunosuppressive therapy driving EBV reactivation—is plausible. Thus, EBV should be described as an IBD-associated virus with pathogenic potential rather than an established driver of IBD progression.

### 4.2. Cytomegalovirus

Cytomegalovirus (CMV) is a ubiquitous DNA herpesvirus that usually causes asymptomatic infection but can reactivate in immunocompromised hosts. In severe IBD, CMV reactivation is particularly frequent in steroid-resistant patients with colonic involvement, especially UC [68,69,70]. Reported prevalence varies widely, with some studies reporting up to 17.3% in patients undergoing colectomy for severe colitis [68]. CMV has been associated with refractory UC and CD, colonic strictures, and worse prognosis in UC [69,70,71]. In DSS colitis mice, CMV can promote IL-23 production through NF-κB activation and exacerbate inflammation [72]. Thiopurine immunosuppression increases the risk of severe CMV infection, potentially through lymphopenia and impaired T-cell responsiveness [73,74]. Pro-inflammatory cytokines such as TNF-α, IFN-γ, and IL-2 may recruit monocytes and dendritic cells that harbor latent CMV, facilitating reactivation [75]. FMT has been reported as a safe and effective therapeutic alternative for CMV colitis in pediatric UC patients, although evidence remains limited [76]. Critically, CMV detection in inflamed mucosa is often cross-sectional; reverse causation—inflammation or immunosuppressive therapy driving viral reactivation—must be explicitly considered. CMV is therefore best interpreted as a marker and potential amplifier of severe mucosal inflammation, rather than a proven primary driver of IBD.

### 4.3. Other Enteric Viruses with Human IBD Relevance

Several other viruses have been associated with IBD or intestinal inflammation. Human norovirus (HuNoV) can increase epithelial permeability and induce aquaporin 1 through NF-κB signaling [77]. Norovirus binds Lewis a and Lewis x antigens on regenerating mucosa in refractory IBD [78]. Hepatitis B virus, hepatitis C virus, varicella zoster virus, human papillomavirus, and SARS-CoV-2 have been reported in IBD cohorts, often in the context of immunosuppressive therapy, malignancy risk, or systemic infection [79,80,81,82,83,84,85]. However, these associations are heterogeneous and should not be grouped with direct virus–microbiota–barrier mechanisms. SARS-CoV-2 infection is associated with gastrointestinal symptoms and altered gut bacterial composition [85,86,87]. Viruses with clinical associations in human IBD are summarized in Table 1.

### 4.4. Animal and Livestock Viruses as Mechanistic Barrier-Disruption Models

Several animal or livestock viruses have been used to study barrier-disruption mechanisms, including porcine astrovirus type 4 (PAstV-4), porcine deltacoronavirus (PDCoV), duck-origin goose parvovirus (D-GPV), transmissible gastroenteritis virus (TGEV), avian leukosis virus subgroup J (ALV-J), and mouse hepatitis virus-1 (MHV-1) [89,90,91,92,93,94,95]. These models have provided valuable insight into TJ disruption, mucus impairment, goblet cell loss, and inflammatory signaling. For example, PAstV-4 activates ERK/MLCK phosphorylation and downregulates MUC2, occludin, and ZO-1 [93]. PDCoV inhibits goblet cell differentiation via Notch signaling [94]. D-GPV increases permeability and alters microbiota through LPS/NF-κB activation [90]. TGEV induces barrier injury via TLR3/TLR4/RIG-I/NF-κB signaling [95]. However, these viruses are not established human IBD pathogens. They should be explicitly labeled as experimental models used to study barrier-disruption mechanisms, not as evidence for human IBD causality. Animal and livestock viruses used as mechanistic barrier-disruption models are summarized in Table 2.

### 4.5. Bacteriophages and the Phageome: The Most Direct Virus–Bacteria Interface

Bacteriophages are the most abundant biological entities in the gut and are arguably the most direct mediators of virus–bacteria interactions. Phages shape bacterial communities through strain-specific predation, lysogeny, and horizontal gene transfer, thereby influencing bacterial metabolism, virulence, and immune activation [96,97]. In IBD, phage populations undergo marked compositional shifts, including expansion of *Caudovirales*, which may disrupt gut homeostasis and impair immune and barrier function [98]. Importantly, transplantation of bacteriophages from ulcerative colitis patients into human microbiota-associated mice shifts the gut bacteriome and exacerbates DSS colitis, providing evidence that phages can move beyond simple association and influence disease severity [99]. Phages can also alter bacterial metabolite production, including SCFAs, which are critical for tight junction protein expression and barrier integrity [100]. Conversely, phage therapy has shown protective effects: bacteriophage EK99P-1 alleviates enterotoxigenic *Escherichia coli* K99-induced barrier dysfunction and inflammation [101]. These findings suggest that phages are not passive bystanders but active regulators of the virome–bacteria–barrier axis. Future studies should prioritize longitudinal sampling, phage–bacteria pairing, and intervention experiments to determine whether specific phages drive or ameliorate IBD-associated barrier injury.

## 5. Mechanistic Framework of Virus–Microbiota Interactions

### 5.1. Definitions and Evidence Framework

Virus–microbiota interactions can be classified into three categories. First, direct viral effects occur when viruses infect epithelial or immune cells and alter barrier function independently of bacteria. Second, microbiota-mediated effects occur when viral infection changes bacterial or phage communities, which then affect barrier integrity. Third, bidirectional interactions occur when viruses alter bacteria and bacteria or their products alter viral infection, stability, or pathogenesis. Throughout this section, we specify which category is supported by the cited evidence.

### 5.2. Phage-Mediated Remodeling of Bacterial Communities

Phages modulate bacterial abundance and diversity through predation, lysogeny, and horizontal gene transfer [96]. In IBD, phage compositional shifts may alter bacterial metabolite production, including SCFAs, thereby affecting tight junction protein expression and barrier integrity [99,100]. Fecal virus-like particles from UC patients can exacerbate DSS colitis in human microbiota-associated mice, indicating that phages may directly or indirectly influence disease severity [99]. Phage EK99P-1 protects against ETEC K99-induced barrier dysfunction and inflammation, demonstrating that specific phages can be beneficial [101]. These findings identify phage–bacteria interactions as a central and potentially causal axis in IBD.

### 5.3. Bacteria Promote Enteric Viral Infection

Gut bacteria can promote enteric viral infection through direct binding to viral particles, enhancement of viral stability, and modulation of host immunity [102,103,104,105,106,107,108,109,110]. For example, poliovirus infectivity increases when it binds to bacterial surface polysaccharides, improving viral particle stability and cellular attachment [105,106]. HuNoV infection of B cells is facilitated by enteric bacteria, and mouse norovirus replication in vivo is influenced by gut bacteria [107]. Type 2 cytokines that stimulate tuft cell growth enhance murine norovirus infection [108]. Bacterial envelope components also enhance reovirus thermostability and intestinal infection [109]. These mechanisms illustrate how bacteria can act as cofactors for viral infection.

### 5.4. Microbiota and Metabolites Inhibit Viral Infection

**Figure 1:**
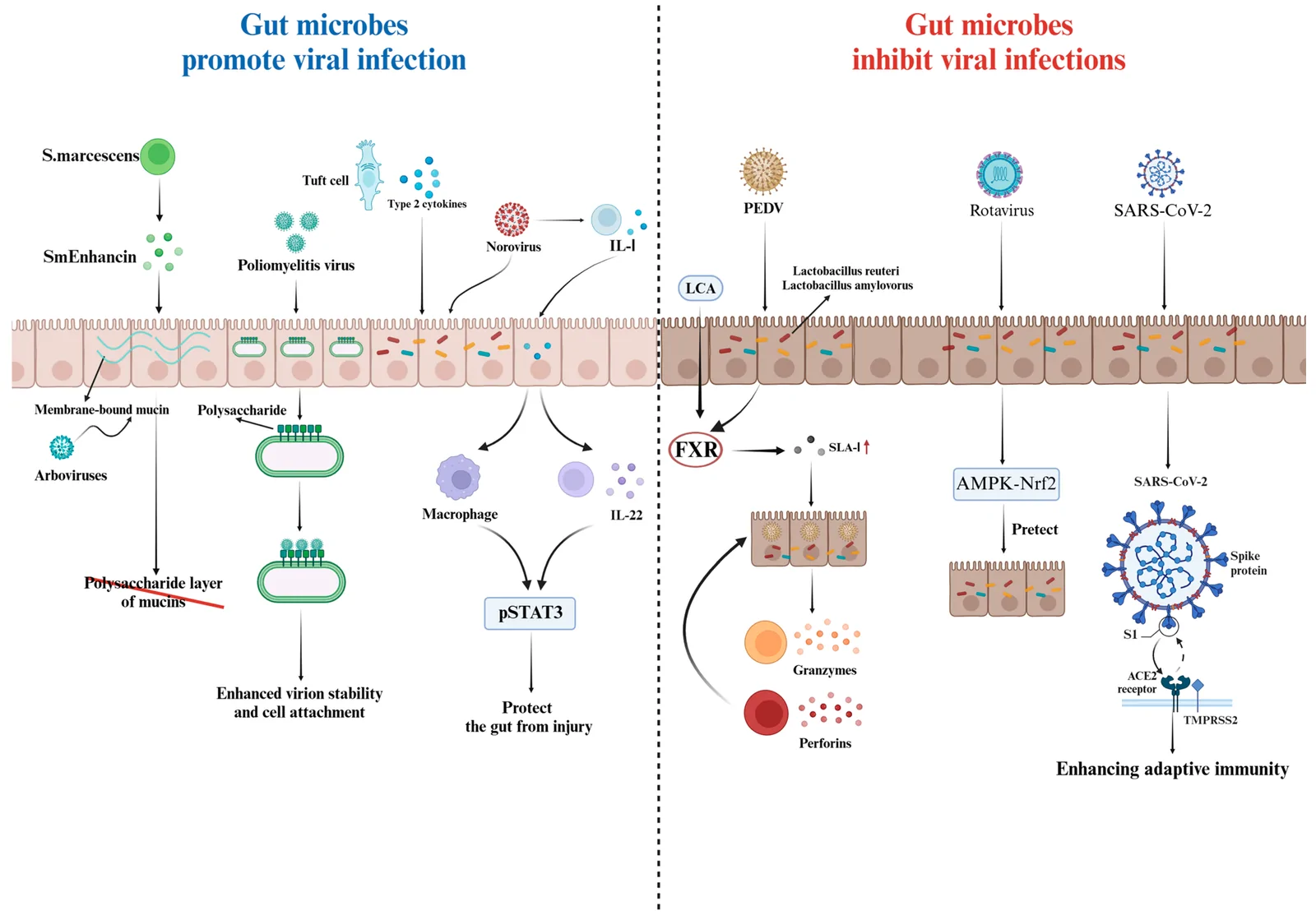
Gut microbes play a dual role in viral infections, both promoting and inhibiting them. The left section *Gut Microbes Promote Viral Infection* shows that *S. marcescens* and *SmEnhancin* promote viral infection by enhancing viral stability and cell adhesion. In addition, they promote viral transmission by increasing immune responses (e.g., production of IL-1 and IL-22). The right section *Inhibition of Viral Infection by Gut Microbes*, on the other hand, demonstrates that microbes such as *Lactobacillus reuteri* and *Lactobacillus amylovorus* inhibit viral infection through activation of the FXR pathway and enhancement of the immune response (e.g., increase in the functioning of swine leukocyte antigens (SLAs) and specific immune cells).

Conversely, gut microbes and their metabolites can inhibit viral infection. Following porcine epidemic diarrhea virus infection, *Lactobacillus reuteri* and *Lactobacillus amyloliquefaciens* enhance SLA-I production via the FXR pathway, facilitating cytotoxic T lymphocyte recruitment and disease regression [111]. SCFAs protect against SARS-CoV-2 by downregulating ACE2 and enhancing adaptive immunity [112]. Microbial metabolites such as nicotinamide oxidation products and specific bacterial strains protect against HSV-1 and SARS-CoV-2 [113,114]. Sodium butyrate protects against rotavirus-induced epithelial barrier damage via AMPK–Nrf2 signaling [115]. NLRP3 and linear enterobactin also modulate antiviral responses [116,117]. These studies suggest that precise microbial or metabolite-based interventions could enhance antiviral immunity, although clinical translation remains challenging. The dual promoting and inhibitory roles of gut microbes in enteric viral infection are summarized in Figure 1.

### 5.5. Viral Infection Perturbs Bacterial Communities

**Figure 2:**
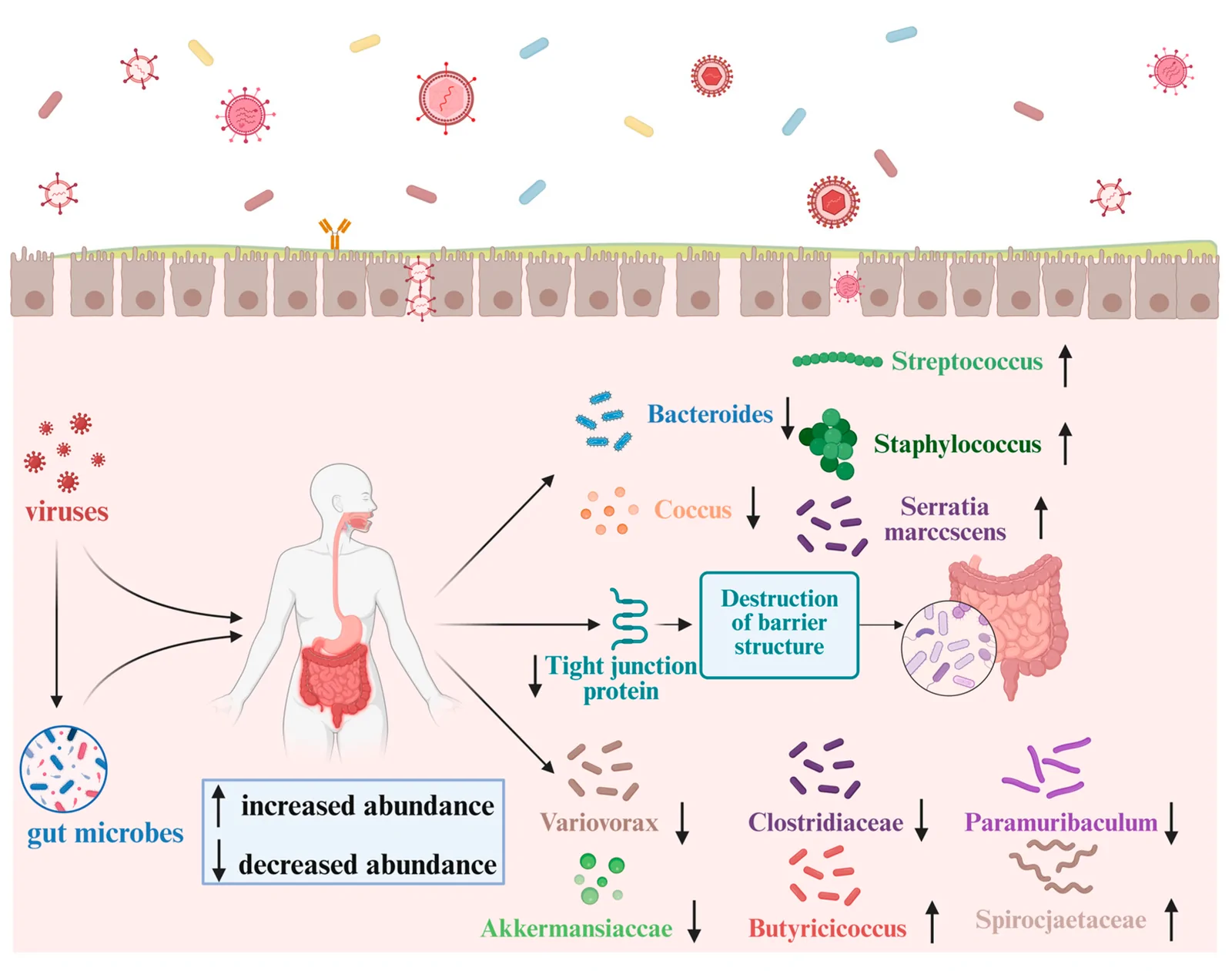
Viral infections lead to disruption of the gut microbial community, which in turn affects the gut barrier structure. Viruses disrupt the function of tight junction proteins by interacting with gut epithelial cells, leading to disruption of the gut barrier structure. The figure shows the changes in different microorganisms, with a decrease in the abundance of *Bacteroides* and *Coccus*, and an increase in the abundance of *Streptococcus*, *Staphylococcus*, and *Serratia marcescens* after virus infection. In addition, the abundances of *Variovorax* and *Clostridiaceae* decrease and the abundances of *Akermansiaceae*, *Butyricoccus* and *Spirochaetaceae* increase, changes indicative of an imbalance in the gut bacteria.

Viral infections can alter gut bacterial composition. SARS-CoV-2 infection is associated with decreased dominant commensal families and increased pathogenic bacteria such as *Enterococcus*, *Staphylococcus*, *Streptococcus*, and *Serratia* [86,87]. However, the specific mechanisms linking these changes to the gut–lung axis remain unclear. Influenza A virus infection reduces tight junction protein expression in lungs and intestines and alters gut microbiota [118]. HIV infection in infants reduces gut microbial α-diversity [119]. Rotavirus infection alters intestinal microbial communities in suckling mice [120]. EBV infection in tree shrews alters gut microbiota and metabolome profiles [121]. D-GPV infection alters microbiota and mucosal barrier function in ducks [90]. ALV-J and PDCoV infections also change microbiota diversity [91,92]. Collectively, these studies indicate that viral infection can disrupt the gut microbial community, which may in turn compromise the intestinal barrier (Figure 2). These studies are limited by sample size and model extrapolation.

## 6. Virus–Microbiota Interactions and Intestinal Barrier Dysfunction in IBD

### 6.1. Direct Viral Effects on Epithelial Junctions and Mucus

Several viruses directly impair epithelial junctions and mucus. PAstV-4 infection activates ERK and MLCK phosphorylation and downregulates MUC2, occludin, and ZO-1 in Caco-2 cells [93]. CMV infection damages AJs and TJs in polarized epithelial cells and increases intestinal permeability in immunocompetent mice, allowing commensals and antigens to translocate to the lamina propria [122]. PDCoV infection stimulates Notch signaling, impeding goblet cell development and mucus secretion [94]. HuNoV increases epithelial permeability and induces aquaporin 1 via NF-κB signaling [77]. D-GPV infection increases plasma D-(−)-lactic acid and LPS and reduces Claudin-3 expression, leading to heightened mucosal permeability [90]. These findings demonstrate direct viral effects on barrier components, although many rely on animal or in vitro models and should not be overgeneralized to human IBD.

### 6.2. Microbiota-Mediated Effects of Viral Infection

Viral effects on barrier function can also be mediated by microbiota. Phages can modify bacterial metabolite production by lysing bacteria, thereby influencing SCFA levels, which are critical for tight junction protein expression and barrier integrity [99,100]. Viral effects on bacteria can indirectly interfere with this process, particularly when virus-mediated bacterial lysis alters the equilibrium of gut bacterial communities, reducing SCFAs and impairing the acellular barrier [100]. Bacteria can also counteract viruses by secreting extracellular polysaccharides that bind viral particles and affect adhesion to epithelial cells [123]. TLR recognition of viral and bacterial PAMPs can trigger inflammatory factor release and destabilize the acellular barrier [124]. Diet-induced alterations in viral–microbiota interactions may further influence barrier function and IBD development [125]. Wnt/β-catenin signaling and the neuroendocrine system may also mediate these interactions [126,127]. However, direct evidence that microbiota mediates viral barrier injury remains limited and requires intervention studies.

### 6.3. Bidirectional Virus–Bacteria Interactions

Bidirectional interactions are best illustrated by phages. Transplantation of bacteriophages from UC patients shifts the gut bacteriome and exacerbates DSS colitis, indicating that phages can influence disease severity [99]. Conversely, phage EK99P-1 alleviates ETEC K99-induced barrier dysfunction and inflammation [101]. Bacteria can also enhance viral infection: bacterial polysaccharides stabilize poliovirus and enhance cellular attachment [105,106]; enteric bacteria promote human and mouse norovirus infection of B cells [107]; and bacterial envelope components enhance reovirus thermostability [109]. These examples show that virus–bacteria interactions are reciprocal and can directly affect barrier function. Future studies should use longitudinal sampling and phage–bacteria pairing to establish directionality.

### 6.4. Immune Activation and UC/CD Differences

Virus–microbiota interactions also shape mucosal immunity. SARS-CoV-2 infection promotes galectin-9 expression in CD4+ T cells by reducing CEACAM5 in enterocytes, increasing inflammatory factor release and CD4+ T cell apoptosis via PI3K/Akt/mTOR inhibition [128]. Hepatitis B virus X protein affects gut mucosal barrier integrity by influencing immune cell transcriptional states in UC patients [129]. CMV and MCMV infections worsen gut inflammation and increase chemokine ligands or pro-inflammatory cytokines [72,75,122,130,131]. TGEV induces inflammatory responses and barrier damage via TLR3, TLR4, and RIG-I-mediated NF-κB signaling [95]. D-GPV infection is associated with altered gut bacteria, particularly Unclassified_S24-7 and *Streptococcus* [90]. Importantly, UC and CD differ in immune phenotype: CD is predominantly Th1/Th17, whereas UC shows a more atypical Th2-like profile [132,133]. Therefore, virus–immune–barrier findings should specify UC or CD rather than generalizing across IBD.

### 6.5. Dysbiosis-Driven Amplification of Barrier Injury

**Figure 3:**
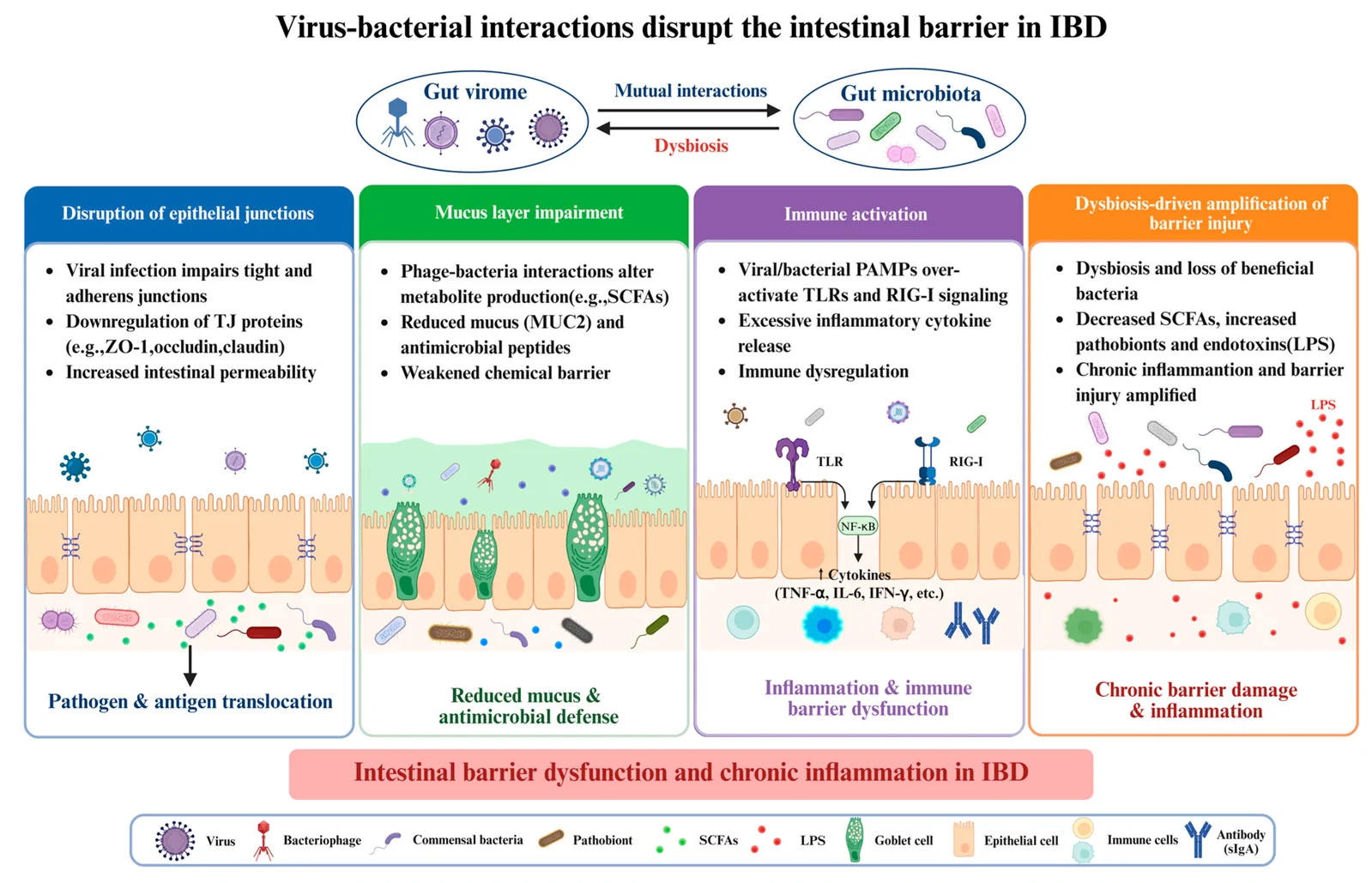
Schematic overview of how virus–bacterial interactions disrupt the intestinal barrier in IBD. Bidirectional interactions between the gut virome and bacterial microbiota regulate intestinal homeostasis under physiological conditions. During IBD, alterations in the virome and microbiota lead to microbial dysbiosis, resulting in disruption of epithelial junctions, impairment of the mucus barrier, aberrant immune activation, and dysbiosis-driven amplification of barrier injury. These interconnected events increase intestinal permeability, reduce antimicrobial defenses, enhance inflammatory cytokine production, and perpetuate chronic mucosal inflammation, collectively contributing to intestinal barrier dysfunction and IBD pathogenesis.

Viral–microbiota interactions can amplify barrier injury through dysbiosis. Infection with *Saccharomyces cerevisiae* strain SD in ducks causes gut structural damage, inflammation, altered microbiota, and reduced SCFAs [134]. In influenza A virus-infected mice, altered gut microbiota and reduced SCFAs are associated with gut damage, inflammation, and impaired barrier function [135]. Phage EK99P-1 prevents ETEC K99-induced barrier dysfunction and attenuates inflammation by modulating osmolality and cytokines [101]. Mouse astrovirus infection alters the mucus barrier and mucus-associated bacteria, affecting resistance to pathogenic *E. coli* colonization [136]. Overall, these findings indicate that virus-bacteria interactions contribute to intestinal barrier dysfunction and IBD pathogenesis through epithelial junction disruption, mucus barrier impairment, aberrant immune activation, and dysbiosis-driven amplification (Figure 3). These findings enhance our understanding of viral–microbiota interactions and offer novel insights for IBD management. Representative studies are summarized in Table 3.

## 7. Conclusions and Future Perspectives

The intestinal barrier is a coordinated system in which epithelial cells, junctional complexes, mucus, AMPs, sIgA, immune cells, and the microbiota maintain homeostasis. Increasing evidence indicates that disruption of this integrated barrier is a defining feature of IBD. Beyond bacterial dysbiosis, the gut virome has emerged as an important regulator of intestinal barrier function through direct infection of epithelial cells, modulation of mucosal immunity, and interactions with bacterial communities. However, most current studies describe associations rather than causality. The key unresolved question is whether virome alterations drive intestinal inflammation or are a consequence of inflammation and immunosuppressive therapy.

Several specific gaps remain. First, it is unclear which viruses can serve as biomarkers in IBD and whether they differ between UC and CD. Second, the most actionable virus–bacteria–host interactions have not been defined. Third, phage–bacteria interactions remain understudied despite being one of the most direct forms of virus–microbiota interaction. Fourth, human evidence is limited, and many mechanistic insights come from animal or in vitro models that cannot be directly extrapolated to human IBD. Fifth, therapeutic strategies such as FMT, phage therapy, engineered probiotics, microbiota-derived metabolites, antiviral interventions, and virome engineering remain early-stage, and current clinical evidence is thin.

Future research should integrate longitudinal clinical cohorts with multi-omics approaches, including metagenomics, metatranscriptomics, metabolomics, proteomics, epigenomics, single-cell sequencing, and spatial transcriptomics. Such approaches should characterize spatiotemporal interactions among viruses, bacteria, host cells, and the intestinal barrier. Intervention studies, including phage transplantation, microbiota depletion, and metabolite supplementation, are needed to establish causality. A more comprehensive understanding of virus–bacteria interactions will accelerate the development of precision medicine for IBD, but therapeutic claims should remain tempered until robust clinical evidence is available.

## Figures and Tables

**Table 1:** Viruses clinically implicated in human IBD.

Virus	Genome	Main Finding Relevant to IBD	Evidence Type	IBD Subtype	Reference
CMV	DNA	CMV reactivation is associated with severe/refractory colitis, especially UC; may amplify mucosal damage	Human association; mouse model	UC > CD	[70,72,75,88]
EBV	DNA	EBV DNA detected in intestinal mucosa of IBD patients in one cohort; associated with lymphoproliferative disorders	Human association; in vitro; mouse	IBD, immunosuppressed	[60,61,62,63,64,65,66,67]
HuNoV	RNA	Increases epithelial permeability via NF-κB/AQP1; binds regenerating mucosa in refractory IBD	In vitro; human tissue	IBD	[77,78]
HBV	DNA	Associated with liver fibrosis and management challenges in IBD	Human cohort	IBD	[81]
VZV	DNA	Immunosuppressants for IBD increase VZV infection risk	Human cohort	IBD	[82]
HCV	RNA	Anti-TNF therapy and interferon therapy complicate IBD management	Human cohort	IBD	[83]
HPV	DNA	Increased incidence/progression in IBD patients treated with azathioprine	Human cohort	IBD	[84]
SARS-CoV-2	RNA	Associated with gastrointestinal symptoms and altered gut bacterial composition	Human cohort/meta-analysis	IBD/COVID-19	[85,86,87]

**Table 2:** Animal/livestock viruses used as barrier-disruption models.

Virus	Host/Model	Barrier Component Affected	Proposed Mechanism	Relevance to Human IBD
PAstV-4 [93]	Caco-2 cells	Mucus and epithelial barrier	ERK/MLCK; MUC2, occludin, ZO-1	Mechanistic model only
PDCoV [94]	Piglets	Mucus barrier	Notch signaling; goblet cell loss	Mechanistic model only
D-GPV [90]	Goose	Tight junctions and microbiota	LPS/NF-κB	Mechanistic model only
TGEV [95]	Piglets/IECs	Immune barrier	TLR3/TLR4/RIG-I/NF-κB	Mechanistic model only
ALV-J [91]	Chicken	Microbiota diversity	Not fully defined	Mechanistic model only
MHV-1 [89]	Mouse	Epithelial integrity	Mucosal inflammation, villous atrophy	Mechanistic model only

**Table 3:** Effects of viruses in experimental models of intestinal inflammation.

Virus	Experimental Model	Major Findings	Barrier Component	Proposed Mechanism	Human IBD Relevance	Evidence Type	IBD Subtype
MCMV/CMV [75,122]	DSS colitis mouse; epithelial cells	Aggravated colitis, increased cytokines, bacterial translocation; increased permeability	Epithelial; immune	TNF-α, IFN-γ, chemokines; junctional disruption	Potential amplifier in severe IBD	Experimental	UC > CD
MuAstV [137]	Mouse	Altered mucus-associated microbiota; increased colonization resistance	Mucus	Mucus ecosystem remodeling	Indirect relevance	Experimental	Not specified
PAstV-4 [93]	Caco-2 cells	Reduced MUC2, ZO-1, occludin	Mucus and epithelial	ERK/MLCK activation	Mechanistic model only	In vitro	Not applicable
PDCoV [94]	Piglets	Goblet cell loss, mucus reduction	Mucus	Notch signaling	Mechanistic model only	Animal	Not applicable
HuNoV [77]	IECs	Increased epithelial permeability	Tight junctions	NF-κB/AQP1	Human-relevant	In vitro	IBD
TGEV [95]	Piglets/IECs	Barrier injury with inflammatory activation	Immune	TLR3/TLR4/RIG-I/NF-κB	Mechanistic model only	Animal/in vitro	Not applicable
D-GPV [90]	Goose	Increased permeability and microbiota alteration	Tight junctions and microbiota	LPS/NF-κB	Mechanistic model only	Animal	Not applicable

## Data Availability

No new data were created or analyzed in this study. Data sharing is not applicable to this article.
